# Chronic Pain: The Extra Burden on Canadian Women

**DOI:** 10.1186/1472-6874-4-S1-S17

**Published:** 2004-08-25

**Authors:** Marta Meana, Robert Cho, Marie DesMeules

**Affiliations:** 1 Department of Psychology, University of Nevada, Las Vegas, USA, 89154-5030; 2 Centre for Chronic Disease Prevention and Control, Health Canada, 120 Colonnade Rd, Ottawa, Canada; 3 Centre for Chronic Disease Prevention and Control, Health Canada, 120 Colonnade Rd, Ottawa, Canada

## Abstract

**Health Issue:**

Chronic pain is a major health problem associated with significant costs to both afflicted individuals and society as a whole. These costs seem to be disproportionately borne by women, who generally have higher prevalence rates for chronic pain than do men.

**Key findings:**

Data obtained from 125,574 respondents to the Canadian Community Health Survey (2000–2001) indicated that 18% of Canadian women suffered from chronic pain, compared to 14% of men. This gender discrepancy, however, seemed to be linked primarily to differences in age, income, and education between adult men and women in this large sample. Age, income, depression and functional interference with activities were strongly associated with chronic pain in general. No gender differences were found in the intensity of pain experienced. Ethnicity was not strongly associated with chronic pain prevalence, although Asians were the group with the highest chronic pain prevalence in the over-65 age group and Aboriginal Canadians had the highest prevalence in the under-65 age group.

**Data Gaps and Recommendations:**

Current gaps in our knowledge include the types of chronic pain women experience, their impact on domestic responsibilities and parenting and health care utilization patterns of women with chronic pain. Data sources such as provincial databases of billing claims may be useful in the future to enrich our knowledge of health care utilization and analgesic medication use. Enhanced surveillance, assessment, and early identification of pain disorders are recommended to improve outcomes. Considering current demographic patterns toward an older population, there is also some urgency to the development of patient education and self-management programs.

## Background

Chronic pain is a major public health problem that places serious stress on afflicted individuals, the health care system and private industry. It has been associated with deficits in quality of life and psychological adjustment, disability, reduced income potential, high levels of health care utilization and high costs to private industry. Generally defined as any continuous or persistent intermittent pain experienced for a period longer than three months,[1] chronic pain affects individuals of all ages and ethnic backgrounds as well as both sexes. However, epidemiologic, clinical and experimental studies have all consistently found that the burden of pain is greater for women than for men.

The magnitude of the sex difference in pain[2,3] is difficult to determine, as it varies, depending on the type of pain and the population being studied. Recent reviews, however, report that the prevalence of most pain conditions is higher among women than men.[4,5] Identifying the sources of this difference in pain is a complex matter that requires a bio-psychosocial perspective.

In terms of biological factors, the transmission and modulation of pain signals may differ in men and women.[5,6] Normal hormonal variations and changes related to women's reproductive functions can be sex-specific sources of pain, as can pathologic processes associated with these.[7] Psychologically, women may differ in their cognitive and emotional processing of pain and also behave differently when in pain.[4] Socially, women differ from men in their societal, family and occupational roles (e.g. multiple primary-role responsibilities), and these may also be potential sources of sex differences in pain.

### Patterns of Pain Prevalence in Canada

In a random survey of 500 households in Ontario, prevalence rates of chronic pain were found to be 11% among people under 60 years and 25% to 40% for those over 60.[8] The 1994–1995 NPHS indicated that 17% of the total population aged 15 and over experienced chronic pain. The prevalence was higher among women than men (20% versus 16%) and increased with age.[9] The most common chronic pain conditions were back pain and arthritis/rheumatism. A survey of 410 adults in the Edmonton area found a prevalence rate of 44%, the most common pain locations being the back, head and neck. Overall, the prevalence was, again, higher among women (65.5% versus 34.5%) but, in this sample, was unrelated to age.[10]

Recent chronic pain prevalence rates in other Western countries are comparable to those found in Canada. U.S. estimates have placed the prevalence rates among women in the United States at 14.7% in the 18 to 50 age range.[11] An Australian survey reported a rate among women of 20% in comparison with 17% among men.[12] In Europe, a Swedish survey found that 23.9% of its sample reported chronic regional pain.[13] In a population-based study in Scotland involving 4,611 individuals aged 25 and over, the prevalence rate of "significant chronic pain" was 14.1% and was higher among women and older age groups.[14]

### Individual Factors Associated with Chronic Pain

Age and socio-economic variables have been associated with chronic pain. For certain pain syndromes, such as joint pain, chronic widespread pain and fibromyalgia, prevalence rates increase with age.[15] Not surprisingly, therefore, chronic pain is also associated with multiple comorbid conditions.[9] As well, some studies have consistently found an association between chronic pain and lower educational levels and socio-economic status.[12,13,16] Psychological distress is common in both men and women who experience chronic pain, depression being a common, strong correlate.[7,17] Estimates of depression prevalence among patients with chronic pain range from 31% to 100%,[18] and pain complaints in depressed individuals range from 34% to 66%.[19] This is particularly a concern for women, as they suffer from clinical depression at twice the rate of men.

The functional interference of pain is also high, and a whole range of activities are often severely curtailed. Daily chores become difficult, ability to work diminishes, and there is a lower rate of full-time employment. [10-12] Social support can also diminish as friends and family lose patience with a problem that is usually invisible and endless.[20] Individuals with chronic pain can also suffer rejection from health care providers frustrated with their failed attempts to heal and with the dependence on pain medications that their patients commonly show.[11]

### Societal Factors Associated with Chronic Pain

In addition to the burden on the individual, chronic pain also exacts a high cost from society at large and the health care system in particular. It is associated with a loss of productivity, high utilization of health services and substantial health care expenditures. Women in North America have a higher rate of health care utilization than men,[21] and this may be, in part, attributable to their higher rates of pain complaints. Direct medical costs for outpatient visits related to chronic pelvic pain alone have been estimated at $881.5 million per year in the United States. Among 548 employed respondents in one study, 15% reported time lost from paid work and 45% reported reduced work productivity.[11] The economic cost of chronic pain to society is very difficult to calculate as it involves various sectors, both public and private. However, judging from the high prevalence rates, high health care utilization by this population, absenteeism, disability, high levels of medication dependence, and the failure of multiple and frequent expensive medical procedures, the economic costs are undoubtedly astronomical.

Pain, which may be a disorder in itself rather than simply a symptom of an underlying condition, is increasingly recognized as a substantial public health problem. More comprehensive and gender-sensitive information on pain is needed in Canada so that enhanced interventions can be developed. In this chapter, the overall burden of chronic pain among Canadian women as well as its determinants and impact are assessed using currently available population health data.

## Methods

Data obtained from the Canadian Community Health Survey (CCHS) Cycle 1.1 (2000–2001) were used in this chapter. This survey was cross-sectional in design and had a total of 131,535 respondents. Health Canada had access to data on the 95.5% (125,574) of these respondents who agreed to share their information. All analyses presented in this chapter used the sample of respondents who agreed to share their information. The prevalence and intensity of chronic pain were compared between men and women and among subgroups of women. Chronic pain status was determined by participants' response to the question "Are you usually free from pain or discomfort?" Those who answered "no" were considered to have chronic pain. The estimated prevalences were calculated using a weighted method to account for the complex sampling design of the survey. The relative contributions of physical/medical (presence of chronic condition(s), etc.) and socio-economic factors to the sex and gender differences were examined using bivariate and multivariate (logistic regression) analysis.

Correlates of chronic pain, including depression, restrictions in daily activity, health care utilization and medication use, were also examined and compared between men and women and subgroups of women.

Statistics Canada's Bootstrap program was used to determine statistically significant differences between prevalence rates for all confidence intervals (CI) reported for the difference between females and males.

## Results

### Prevalence of Chronic Pain

**Figure 1:**
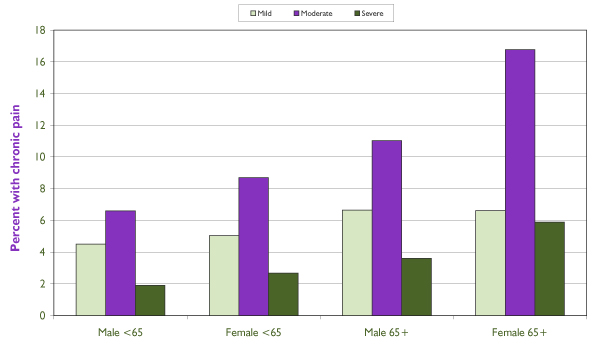
**Chronic Pain Prevalence by Age, Sex and Intensity, Canada. **Source: Statistics Canada, CCHS, 2000–2001

According to data collected from Cycle 1.1 (2000–2001) of the CCHS, 16% of the population 12 years of age and older suffered from chronic pain (14% males versus 18% females, 95% CI 3.73, 4.99). Classification of pain as either mild, moderate or severe was proportionally similar in males and females (Figure 1).

**Figure 2:**
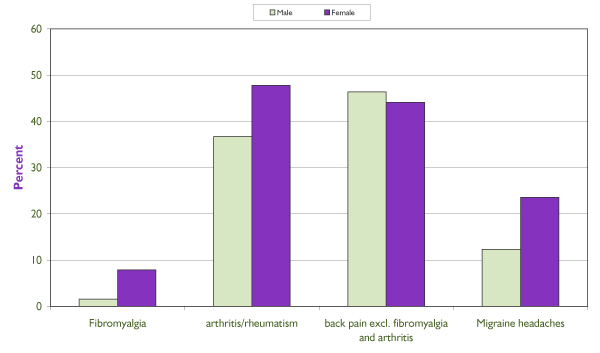
**Prevalence of Selected Chronic Conditions Among Those With Chronic Pain, by Sex, Canada. **Source: Statistics Canada, CCHS, 2000–2001

Although many conditions can result in chronic pain, the survey asked specifically about four conditions known to be strongly related to chronic pain. Among those with chronic pain in this study, the prevalence of arthritis/rheumatism (95% CI 9.29, 12.97), fibromyalgia (95% CI 5.51, 7.06) and migraine headaches (95% CI 9.76, 12.68) is significantly higher among women than men (Figure 2). There is a slight difference in the prevalence of back pain (95% CI 0.20, 4.27) among those who report chronic pain (excluding fibromyalgia and arthritis). Although the difference in back pain is statistically significant, the practical implication of this difference warrants further investigation. The prevalence of fibromyalgia is low among those who report chronic pain (Figure 2).

### Individual Factors Associated with Chronic Pain

**Figure 3:**
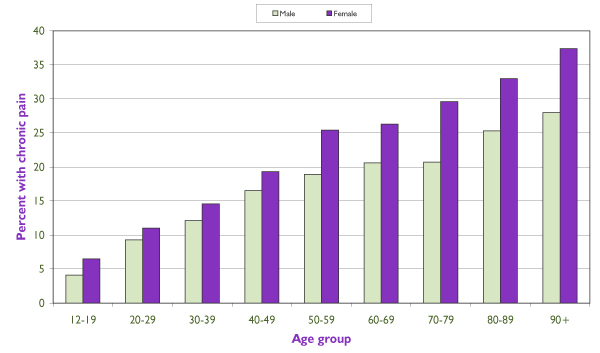
**Prevalence of Chronic Pain by Age and Sex, Canada **Source: Statistics Canada, CCHS, 2000–2001

**Figure 4:**
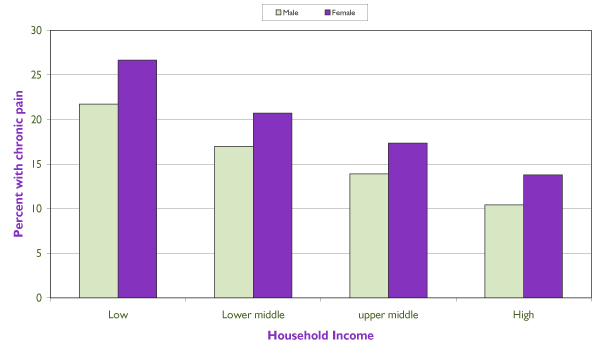
**Chronic Pain Prevalence by Household Income, Canada. **Source: Statistics Canada, CCHS, 2000–2001

The prevalence of chronic pain increased with age in both sexes (Figure 3). The prevalence was higher among females than males at all ages. There was also a clear association between household income and chronic pain (Figure 4). The prevalence of chronic pain was lower among those in higher income categories and higher for those in lower income categories.

**Figure 5:**
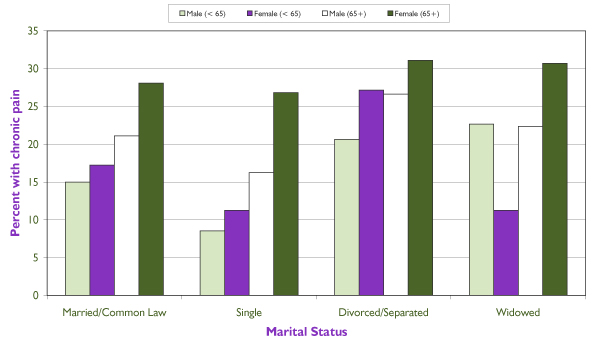
**Prevalence of Chronic Pain by Sex, Age and Marital Status, Canada. **Source: Statistics Canada, CCHS, 2000–2001

Marital status appeared to be associated with chronic pain. In both sexes and for all ages, chronic pain prevalence was lowest among those who were single (Figure 5) and, except among males less than 65 years, highest in those who were divorced or separated. There were no differences in chronic pain prevalence by family structure.

**Figure 6:**
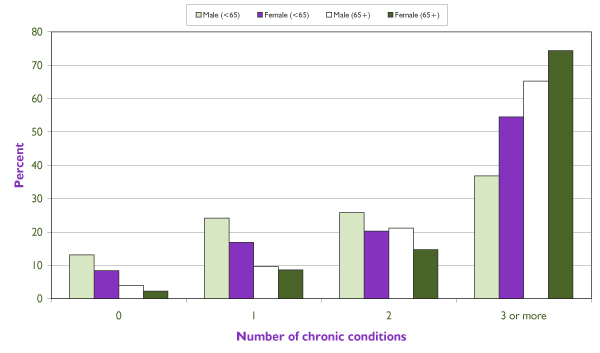
**Distribution of Chronic Conditions Among Those With Chronic Pain, by Age and Sex, Canada. **Source: Statistics Canada, CCHS, 2000–2001

Across age and sex, the majority of those with chronic pain had three or more chronic conditions, whose prevalence increased with age for both sexes (Figure 6).

**Figure 7:**
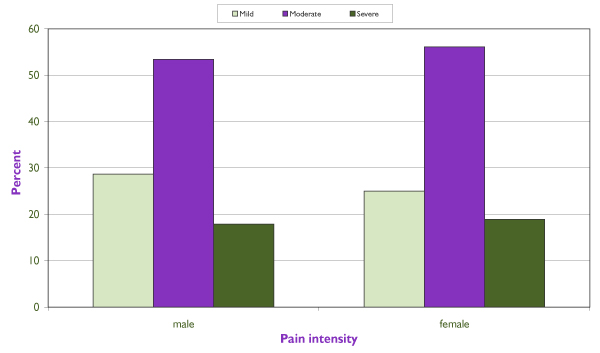
**Pain Intensity Among Those With Pain-Associated Chronic Conditions, Canada. **Source: Statistics Canada, CCHS, 2000–2001

Pain intensity was similar for males and females (Figure 7). Proportionally, this study found that the level of pain intensity among those who suffered from chronic conditions associated with pain (back pain, fibromyalgia, arthritis/rheumatism and migraine headaches) was similar to the level among those suffering from other types of chronic pain.

**Figure 8:**
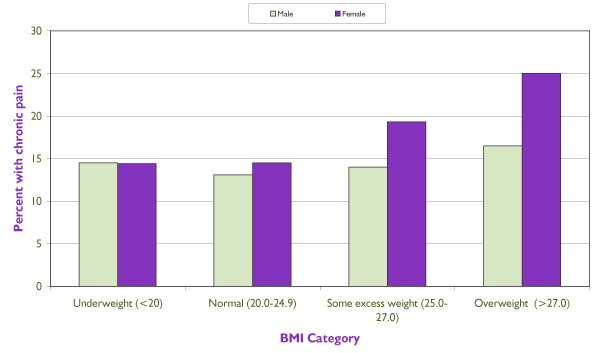
**Chronic Pain Prevalence by Sex and BMI, Canada. **Source: Statistics Canada, CCHS, 2000–2001

A high body mass index (BMI) has been found to be associated with increased mortality and decreased life expectancy. Comparison of chronic pain prevalence across BMI also revealed an association: the prevalence was higher for each subsequent BMI category among females, with the lowest prevalence among those who had a BMI of less than 20 and the highest among those with a BMI of greater than 27 (Figure 8). Among males, the prevalence of chronic pain was similar in all BMI categories. Female chronic pain prevalence was significantly higher than that of males in the "some excess weight" and "overweight" categories (95% CI 3.41, 7.21 and 7.14, 9.92 respectively).

**Figure 9:**
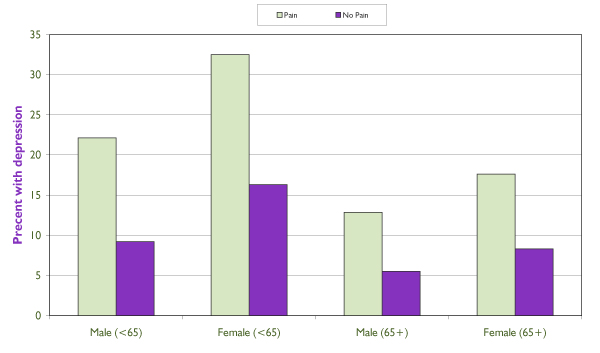
**Prevalence of Depression by Chronic Pain Status, Age and Sex, Canada. **Source: Statistics Canada, CCHS, 2000–2001

The prevalence of depression was twice the rate among those who reported chronic pain as among those who did not and appeared to be related to age for both males and females (Figure 9). The prevalence of depression was almost twice as high among individuals with chronic pain who were aged less than 65 years as among those 65 years and older for both males and females (Figure 9).

**Figure 10:**
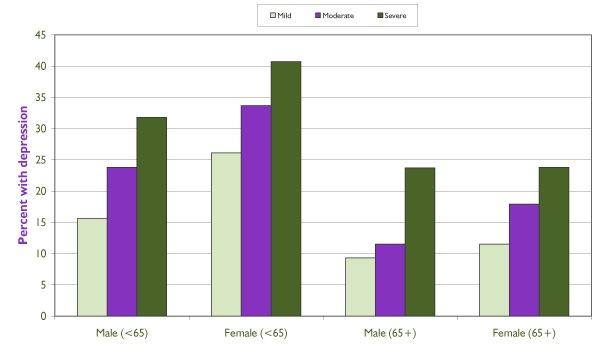
**Prevalence of Depression by Pain Intensity, Age and Sex, Canada **Source: Statistics Canada, CCHS, 2000–2001

Depression was also related to pain intensity for both sexes. Figure 10 shows that a higher level of pain intensity was associated with a higher prevalence of depression.

Chronic pain affects daily tasks and can cause restrictions in daily activities. In both age categories (less than 65, and 65 and older) the majority of those who suffered from chronic pain were limited in at least "a few" activities as a direct result of their pain. The percentage of females who were limited in a few or more activities was higher than the percentage observed among males (77.7% versus 70.7%).

**Figure 11:**
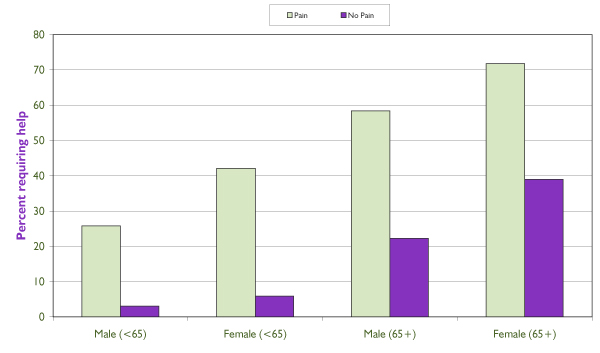
**Proportion Requiring Help by Chronic Pain Status, Sex and Age, Canada **Source: Statistics Canada, CCHS, 2000–2001

Comparing individuals with chronic pain to those without revealed that the proportion requiring help with at least one task was substantially higher among those suffering from chronic pain than those who were free from pain (Figure 11).

**Figure 12:**
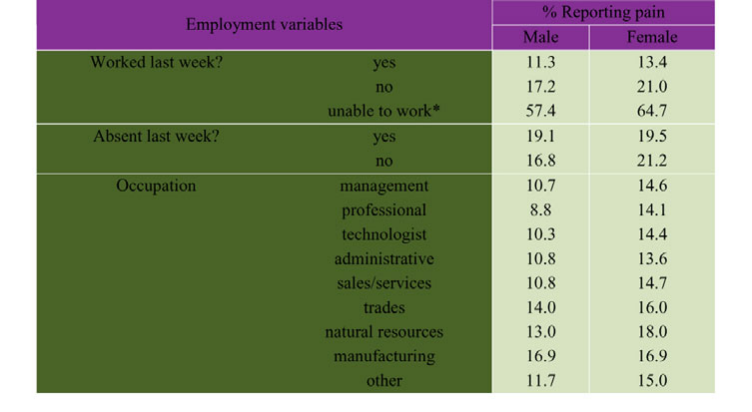
**Prevalence of Chronic Pain by Employment Variables **Source: Statistics Canada, CCHS, 2000–2001

For those who suffered from chronic pain, employment issues were very important. In this sample, it was found that the majority of those who were unable to work in the week before being interviewed suffered from pain (Figure 12). Chronic pain also appeared to be associated with type of occupation, the prevalence being lowest among professionals and highest in occupations in manufacturing and natural resources. The association between type of occupation and prevalence of chronic pain was similar among males and females.

**Figure 13:**
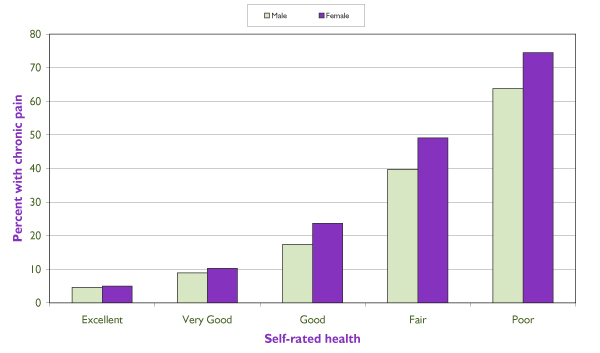
**Prevalence of Chronic Pain by Self-Rated Health, Canada. **Source: Statistics Canada, CCHS, 2000–2001

Poor self-rated health was inversely related to chronic pain (Figure 13). Those who ranked their health as excellent had the lowest prevalence of chronic pain, and the prevalence was highest among those who felt that their health was poor. This trend was similar in both males and females.

**Figure 14:**
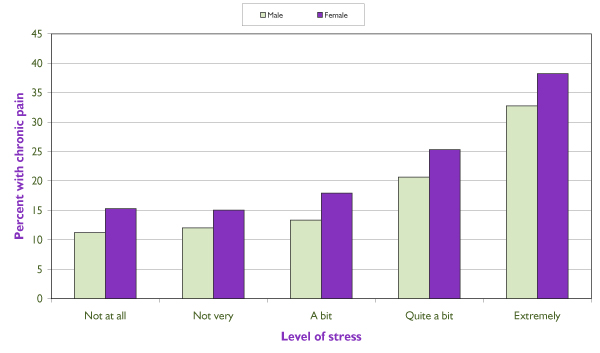
**Prevalence of Chronic Pain by Self-Rated Stress, Canada. **Source: Statistics Canada, CCHS, 2000–2001

Self-rated stress was related to chronic pain, in that those who were extremely stressed had the highest prevalence of chronic pain and those with lower levels of stress had a lower prevalence of chronic pain (Figure 14).

In this analysis, social support was measured by a variable referred to as "tangible social support," which measures whether the individual had somebody to take them to the doctor, do their chores, prepare meals or help if they were confined to a bed.

**Figure 15:**
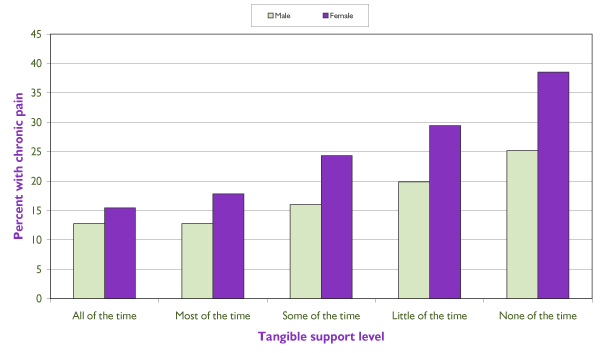
**Prevalence of Chronic Pain by Perceived Tangible Support, Canada **Source: Statistics Canada, CCHS, 2000–2001

Figure 15 shows that there was a negative association between chronic pain and perceived social support. Pain was reported more frequently by those who received less social support. This was true for males and females and indicates that perceived social support is an important factor to consider in those with chronic pain.

**Figure 16:**
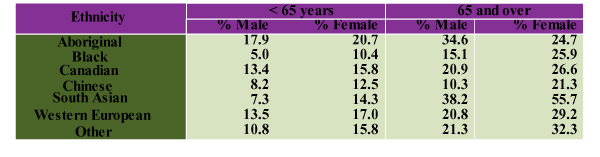
**Percentage of Those With Chronic Pain, by Sex, Age, Ethnicity **Source: Statistics Canada, CCHS, 2000–2001

There do not seem to be major ethnic differences in chronic pain prevalence, with the notable exception of two ethnic groups. For both sexes, in the age group 65 years and older the proportion of South Asians who reported chronic pain was greater than for any other ethnic group. Chinese males and females had the lowest rates for this age group. Among those aged less than 65 years, Aboriginals had the greatest proportion of reported chronic pain, for both sexes (Figure 16).

In bivariate analysis, level of education was not associated with chronic pain for either of the sexes or age groups.

**Figure 17:**
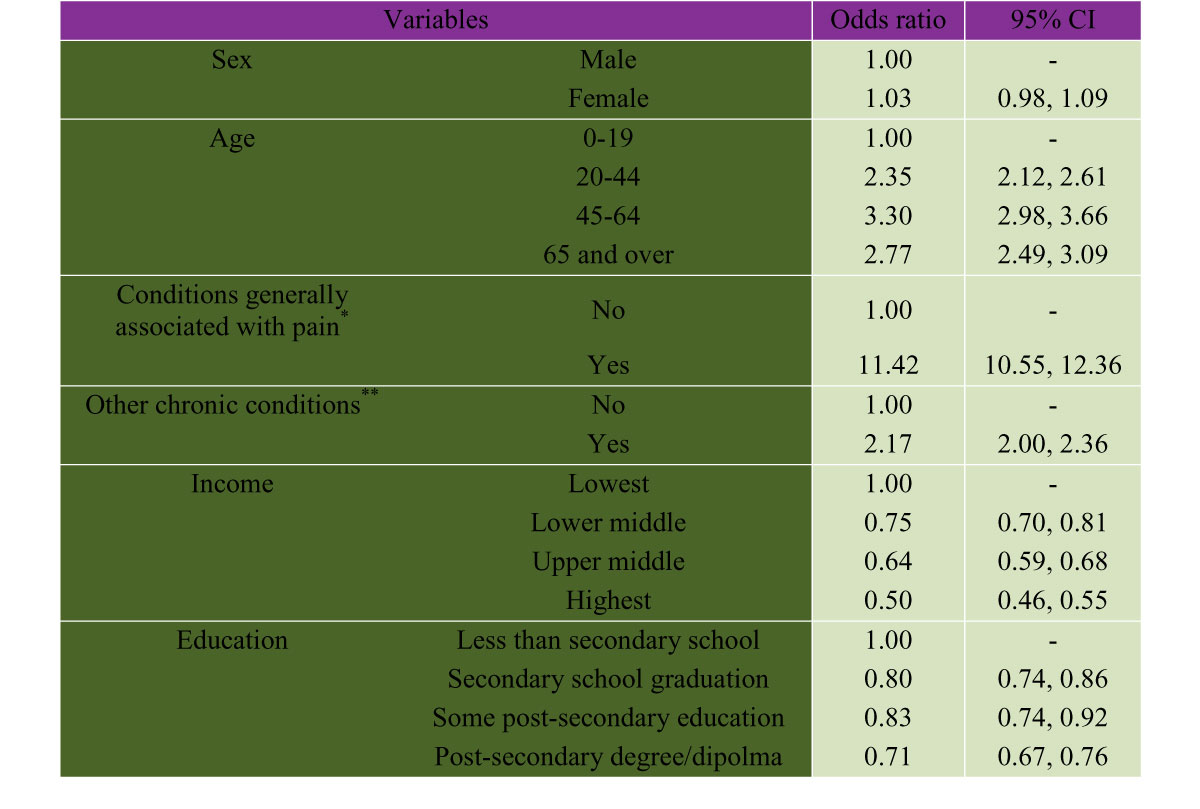
**Logistic Regression of Variables Associated With Pain, Odds Ratios **Source: Statistics Canada, CCHS, 2000–2001. Statistics Canada bootstrap programs used

Figure 17 is a summary table of a multivariate logistic regression model. The results of the regression show that when age, chronic conditions associated with pain, other chronic conditions, income and education were controlled for, females did not have an increased risk of chronic pain as compared with males.

### Societal Factors Associated with Chronic Pain

**Figure 18:**
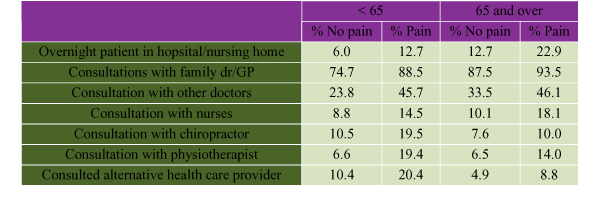
**Percentage Health Care Utilization in The Previous 12 Months, by Chronic Pain Status and Age. **Source: Statistics Canada, CCHS, 2000–2001

Chronic pain sufferers have a substantial impact on the use of health care services. As shown in Figure 18, comparisons for all selected indicators of health care utilization showed that use was higher among those who reported suffering from chronic pain than those who did not. Use of chiropractors, physiotherapists and alternative health care providers was lower among those 65 years and older.

**Figure 19:**
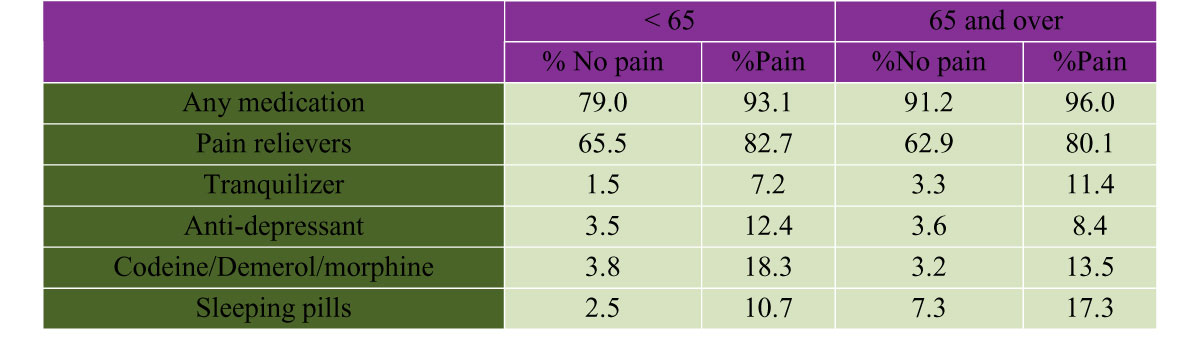
**Medication Use in Previous Month by Chronic Pain Status and Age. **Source: Statistics Canada, CCHS, 2000–2001

Medication use was higher among those reporting chronic pain than those not doing so for all medications in general and all selected types of medication (Figure 19). The use of pain medications such as pain relievers, tranquilizers, antidepressants and opiates was two to four times as high in those with chronic pain than in those without chronic pain.

## Discussion

### Characteristics of Chronic Pain Sufferers

The chronic pain sufferer in Canada is more likely to be a woman than a man, although the gender difference is not tied exclusively to sex. Women also have lower incomes, less formal education and twice the prevalence of depression, all of which were strongly associated with the report of chronic pain in this study. It thus seems reasonable to speculate that the differences in chronic pain evidenced in this CCHS were attributable to a combination of biological and psychosocial conditions specific to each sex. Not surprisingly, chronic pain was also strongly associated with age and multiple chronic conditions. Women with chronic pain were more likely to report fibromyalgia, arthritis and migraine headaches, although there was no significant sex difference in the prevalence of back pain. In terms of potential impact, chronic pain was strongly related to reports of poor health, high levels of stress, low levels of social support, more functional interference with work and other activities, higher levels of dependence on others, higher levels of health care utilization, and higher medication usage.

### Treatment Approach

The "chronic" in chronic pain encapsulates the sense of defeatism that characterizes the common attitude of many patients and health care providers who are dealing with this perplexing and debilitating problem. The etiology of most chronic pain syndromes remains largely unknown and, consequently, treatment efforts have consisted of pain management, at best, and narcotic dependence, at worst. Pain is a multi-dimensional problem not amenable to single causal pathway explanations or treatment approaches. It involves biological processes as well as cognitive, emotional and social ones. Chronic pain thus presents a challenge to both health care providers and patients understandably searching for a quick and definitive solution.

Multidisciplinary treatment is indicated for multi-dimensional problems such as chronic pain. In addition to medical and physical therapy, cognitive-behavioural approaches have been shown to be important components in treatment. Recent research indicates that behavioural interventions are generally superior to medical treatment controls in improving pain, decreasing disability and increasing activity levels.[22] These interventions can also have the effect of teaching patients skills for continued self-care.[23]

### Implications for Health Care Utilization

Despite the demonstrated effectiveness of multidisciplinary approaches to the treatment of chronic pain, it is only a highly select group of patients who ever reach multidisciplinary pain clinics. Most chronic pain patients show a pattern of repeated consultations with primary care doctors and high levels of multiple consultations in the hopes of finding the one who will solve the problem. This high level of consultation is sometimes also fuelled by the search for more prescription analgesics, after the tolerance of prior health care providers has been exhausted. The continuity of care becomes a major problem as patients skip from one provider to another. The elusive treatment is never found, drug dependence is common, and the consequent expense is a major burden on the Canadian health care system.

Although specialty pain clinics are often perceived as expensive ventures, their treatment outcomes can result in lower levels of patient disability. They are thus likely to have an impact on health care utilization.[24] The economics of health care may be such that high front-end investments may result in long-term health care savings for the system as a whole.

### Data Limitations

The data source for the analysis in this chapter was cross-sectional in nature, and as a result causal pathways are difficult to infer. Also, since the survey was based on respondents' self-reports, the quality and accuracy of the data cannot be determined. Furthermore, the survey asked respondents about only four specific chronic conditions that have been associated with chronic pain. Clearly, there are many other conditions that can result in chronic pain.

### Gaps and Recommendations

There are a number of gaps in the chronic pain and gender data currently available. One major gap is the lack of detailed data on the types of chronic pain that women experience. Chronic pelvic pain is an example of a gender-specific pain tied to women's reproductive function for which there is little Canadian, population-based data, despite ample U.S. evidence indicating that this is a major women's health care problem. Endometriosis and polycystic ovarian disease are just two of the common disorders of reproductive function that result in chronic pelvic pain, although much of this pain is without obvious pathology. Vulvar vestibulitis and vulvodynia are also increasingly reported in both pre- and post-menopausal women. Temporomandibular joint disorder (TMJ) is yet another example of a chronic pain disorder that affects predominantly women.

A second major gap in the Canadian literature is systematic data collection from sources other than self-reports. Provincial databases of billing claims need to be investigated to obtain a clearer picture of health care utilization, prevalence of pain disorders and pain disorder-related patterns of analgesic medication prescription. The Pharmacare data from some provinces could be useful in inferring the presence of chronic pain syndromes, as the prevalence of heavy users of analgesic drugs could be detected using this source. In addition, many women report reproductive function-related pains to obstetricians or gynecologists who often serve as their primary care providers. Studies on women's health care need to increase the focus on this group of providers in addition to the existing focus on primary care doctors. Billing databases may also serve to clarify the currently murky picture regarding chronic pain prevalence in different ethnic groups. Cultural differences in the acceptability of reporting pain may be obscuring ethnic trends that could be directly targeted by public health efforts.

A third gap of particular importance to women is the lack of assessment of the functional impact of chronic pain on domestic responsibilities and parenting. The CCHS and many other surveys have consistently shown a connection between reports of chronic pain and employment interference, but there is very little investigation into the impact of pain on work in the home. This kind of functional impairment gets lost in the employment data. If chronic pain is interfering with work outside the home, it is most likely also interfering with work inside the home. The lack of assessment of this type of interference only serves to marginalize an important aspect of women's lives and hide the wide-ranging deleterious effect of chronic pain on women and their families.

Filling these gaps in our knowledge about women and pain is likely to prove integral to the development of strategies designed to reduce the impact of chronic pain. Certain recommendations, however, can already be suggested. Surveillance and early identification of pain disorders is crucial, as there are both theoretical and empirical reasons to believe that early treatment will result in better outcomes. Untreated pain can establish a central nervous system hold that becomes increasingly resistant to peripheral and other interventions. Long-standing pain can also result in behaviour patterns (e.g. lack of activity) that lead to other complicating disorders (e.g. obesity) and to cognitive and emotional problems (e.g. depression) that complicate treatment. Finally, the more long-standing the pain, the more likely is the dependence on narcotics and other pain medications.

Primary care providers and obstetrician/gynecologists are crucial to this surveillance and early detection effort. Assessment of pain needs to be incorporated into the first consultation and targeted, even if it is not the primary reason for the consultation. It then needs to be reassessed periodically. Patient education about chronic pain syndromes is also important, as it can establish hopeful yet realistic expectations and moderate the impulse to consult multiple doctors in search of "the cure." It can also be an efficient way to teach patients self-management strategies that have been empirically shown to lead to significant decreases in pain, disability and medical consultations.[23] As well, women have been shown to be more amenable to this type of self-care than men.

Multidisciplinary pain clinics have demonstrated effectiveness and may, in the long term, be the most economical and effective recourse in the treatment of chronic pain. Rather than envision these as clinics within large, central metropolitan hospitals, perhaps smaller community-based versions would better serve the population in question. These smaller clinics would be more accessible to women who may be older, be disabled, or have lower income and/or have children, and they could also be tailored to the culturally specific characteristics of the community.

## Conclusion

Chronic pain is a daunting problem for both individuals and society. Its effects on quality of life and economic costs demand attention as we enter the twenty-first century and plan for improvements in the delivery of health care to Canadians. The current age structure of the Canadian population indicates a large expected increase in the number of individuals who are over the age of 65 over the next 30 years. This necessarily means an increase in the prevalence of chronic pain, especially among women. Strategies for addressing this growing problem are needed to reduce the overall impact of chronic pain. The collection of more finely gradated information on the nature and impact of chronic pain and health care utilization is necessary, yet health care delivery strategies cannot wait for all of the information to be collected. The aim of this survey and report is to make a contribution to future data collection efforts and to ongoing and future applications centred on the care of both men and women suffering from chronic pain.
